# A sensitive chalcogenide-based electrochemical sensor for ultra-level detection of Mospilan residues in real samples

**DOI:** 10.1038/s41598-025-89256-x

**Published:** 2025-02-18

**Authors:** Amirkhosro Beheshti-Marnani, Tahereh Rohani, Mahdokht Arjmand kermani, Sayed zia Mohammadi

**Affiliations:** 1 Department of Chemistry, Payame Noor University, Tehran, 19395-4697 Iran; 2 Agricultural Engineering Research Department, Kerman Agricultural and Natural Resources Research and Education Center, Agricultural Research, Education and Extension Organization (AREEO), Kerman, Iran

**Keywords:** Bismuthinite, CuS, Mospilan, Modified electrode, Graphene oxide, Chemical tools, Chemical modification, Analytical chemistry, Catalysis, Chemistry

## Abstract

Addressed herein, the synthetic bismuthinite and bismuthinite@copper sulphide as two metal chalcogenides have been applied for modifying a glassy carbon electrode(GCE). The as-prepared nanomaterials were characterized using X-ray diffraction (XRD), scanning electron microscopy(SEM) and Energy-dispersive X-ray spectroscopy(EDX). By comparing the results, bismuthinite @copper sulphide hybridized with graphene oxide (GO) modified electrode exhibited superior sensitivity for detection ultra-levels of pesticide Mospilan (acetamiprid) in real samples. The dynamic concentration range of acetamiprid was found to be 80-680nM with a remarkably low detection limit about 4.1nM along with good stability and repeatability. Finally, the fabricated electrochemical sensor, bismuthinite@copper sulphide/GO, was suggested as a suitable alternative to more complex enzyme-based and aptamer-based methods for Mospilan detection.

## Introduction

Undoubtedly, usage of pesticides and herbicides plays an important role in human food supply. Unfortunately, most of them keep residues in water, soil and crops. According to Stockholm convention, over two-thirds of decomposable organic pollutants belongs to the category of pesticides^1,2^.

Mospilan or acetamiprid, as a neonicotinoid neural active pesticide with chemical formula C_10_H_11_ClN_4,_is effective against the pests across a wide range of agriculture crops^3,4^.

However, the mentioned pesticide has several negative side effects on human body including the destruction of beneficial bacteria and blood lymphocytes, as well as DNA damage^5–7^.

Acetamiprid (Mospilan) has largely replaced organophosphorus pesticides against sucking insects in leafy crops like tea, vegetables and some fruits^8,9^. However, sensitive and accurate detection of acetamiprid, like other pesticides, remains a challenge.

Several techniques has been employed for detection of acetamiprid in various samples like high performance liquid chromatography(HPLC)^10,11^, Liquid chromatography–mass spectrometry(LC-MS)^12^, fluorimetry^13,14^, enzymatic methods^15,16^and gas chromatography^17^. While these are standard techniques, they often suffer from drawbacks such as being time-consuming, requiring complex operation and expensive equipment and reagents, and necessitating difficult sample pretreatment^18,19^. Chemiluminescence and aptamer-based methods offer improved speed and selectivity but remain expensive and labor-intensive^20–23^. Fluorescence techniques often require heavy metals and organic dyes, posing health and environmental risks^24^.As well, Colorimetric methods suffer from low sensitivity and time-consuming sample preparation^25^.

Non-enzymatic electrochemical methods offer a promising alternative for food and crop monitoring and quality control due to their inherent advantages^26,27^Electrochemical sensors based on various carbonaceous materials like fullerene, nanotube, graphene and metal oxides such as zinc oxide were developed for pesticides analysis as modified electrodes^28–31^. Zhao et al. used various modifiers and cyclic voltammetry for the sensitive determination of methyl parathion, employing the Michaelis-Menten constant for analytical purposes^32^.

Differential pulse voltammetry, used by Huo et al. for detecting organophosphorus pesticides with copper oxide nanoparticle-hybridized single-wall carbon nanotubes as the electrode modifier, is another suitable method for pesticide analysis^33^. A cobalt oxide/reduced graphene oxide modified electrode and square wave voltammetry were employed for measuring carbofuran and carbaryl in fruit and vegetable samples^34^.

In recent years, electrochemical impedance spectroscopy (EIS) was applied for detection of organophosphorus pesticides^35,36^. Also, modifiers like CeO_2_-Au/MWCNT^37^and Zirconium nano particles/ graphene were used for detection of methyl parathion by EIS^38^. The literature reveals a wide range of electrode modifier materials for pesticide detection, including carbonaceous materials, metal oxides/sulfides, zeolites, and clays^39–42^. Metal oxides like SnO_2_, TiO_2_, RuO_2_, MgO, V_2_O_5_possessing uniform crystalline structures with diverse morphologies and electrocatalytic properties, have been extensively used in electrochemical sensor development^43,44^. Up to now, parathion^45^carbofural and carbaryl^34^and organophosphorus pesticides^33^ have been detected by metal oxide electrode modifiers.

Metal and transition metal chalcogenides, graphene-like materials such as MoS_2_, WS_2_, MoSe_2_are another class of metallic-based materials used in sensing. These materials are widely used in electronic energy storage and optical sensing. Their sheets interact through van der Waals forces, with a metal atom bonded to two chalcogen atoms, forming triplet layers^46–48^.

Nowadays, a few researchers have been applied metal chalcogenides for pesticide sensing development^49,50^. For example, MoS_2_quantum dot particles was applied for fabricating a label-free fluorescent nanoprobe sensors for detection of methyl parathion^51^.

The high stimulated entropy in multicomponent metal/transition metal chalcogenides is thought to create unique physical properties due to synergistic effects and efficient charge distribution, enhancing electrocatalytic activity^52^. Bulk bismuth has rhombohedral layers with a relatively large interlayer distance (3.1 Å)^53^, allowing the intercalation of various cations (alkaline and transition metals)^54,55^.Moreover, the surface of unitary 2D bismuth has many coordinately unsaturated Bi atoms which could lead to placement many heteroatoms and then electrocatyltic effects^56,57^. Binary 2D bismuth based materials are belonging to p-type semiconductors. It is believed that the mentioned materials have very narrow band gaps(1.5-1.8ev) due to p-state and s-p state which grant high mobility of charge carriers to bismuth chalcogenides^58,59^. However, the incorporation of metallic atoms such as copper reduces the band gap to lower values (1.4 eV)^60–62^. promoting charge transfer and enhancing electrocatalytic effects. Furthermore, hybridizing metal chalcogenides with 2D materials like graphene improves sensing performance by increasing surface area and conductivity^63^.

In the current work, trace levels of remaining acetamiprid as a high consumption pesticide was detected through a simple electrode modified with two metal chalcogenides, bismuthinite (Bi_2_S_3_) and Bi_2_S_3_@CuS. The electrooxidation of acetamiprid proved more favorable than the oxygen release reaction on the surface of the modified glassy carbon electrode (Bi_2_S_3_@CuS/GO-GCE). The bringing results confirmed that the fabricated Bi_2_S_3_@CuS/GO-GCE is a simple and cost effective sensor for ultra-trace analysis of Mospilan in real samples with no need to expensive and complicated implements.

## Experimental

### Chemicals and instrumentals

Cu(NO_3_)_2_.3H_2_O, Bi(NO_3_)_3_.5H_2_O and thiourea, the primary reagents required for nanocomposite synthesis, were purchased from Merck.

Acetamiprid pesticide (purity > 98%), dissolved in dimethyl sulfoxide and graphite powder were supplied by Sigma-Aldrich. Other required reagents were purchased from Merck in their analytical grade.

Phosphate-buffered saline (PBS) was prepared by titrating 0.1 M of phosphoric acid to 0.1 M of NaOH solution.

Cyclic and differential pulse voltammetry studies were performed using a Metrohm 797 VA Computrace. Electrochemical tests were carried out in a three-electrode system including a platinum electrode as a counter (auxiliary) electrode, a silver/silver chloride electrode (in 3 M of KCl) as a reference electrode, and a glassy carbon electrode(GCE) of Metrohm Company with internal diameter of 2 mm as a working electrode.

Panalytical X’Pert Pro MRD X-Ray Diffractometer (the Netherlands) equipped by copper anode was used to prepare the X-ray diffraction.

spectra of the synthesized materials.

### Synthesis of Bi_2_S_3_ nanocomposite

Bismuthinite (Bi_2_S_3_) nanoparticles were prepared via a hydrothermal method. First, 70 ml of a solution containing 0.1 M of Bi(NO_3_)_3_.5H_2_O and 0.15 M of thiourea was prepared. Then, 5 ml of a 65% nitric acid was added to the pervious solution and stirred for 20 min. The resulting mixture was transferred to a stainless steel reactor and heated at 180 ºC for 12 h. The obtained black material was collected and washed twice with deionized (DI)water and ethanol. After that, the product was dried in an oven at 60 ºC. The as-synthesized material was characterized by X-ray diffraction (XRD), energy-dispersive X-ray spectroscopy (EDX), and scanning electron microscopy (SEM) to determine its composition, purity, and morphology.

### Synthesis of Bi_2_S_3_@CuS as a two component metal chalcogenide

In this procedure, a 5.0 g portion of Cu(NO_3_)_2_.3H_2_O was dissolved in 100 ml of DI water. Then 3.0 g of the Bi(NO_3_)_3_.5H_2_O was dissolved in 50 ml of DI water and added to pervious solution. Next, 150 ml of 0.5 M thiourea was transferred to the resulting mixture and stirred for half an hour. Then the solution was conducted to a stainless steel reactor and heated to 190 ºC for a day and night. After cooling, the product was collected by centrifugation and dried in an oven at 60 ºC. Finally, the product was characterized by XRD, EDX, and SEM.

### Synthesis of graphene oxide(GO)

Herein, the modified Hummers method was used for GO synthesis^64^. In summary, 100 ml of an acidic solution with a volume ratio of 9:1 of sulfuric acid and phosphoric acid was slowly added to 5 g of graphite powder. Then, a 5.0 g portion of potassium permanganate was added to the mixture and refluxed at 90 ºC for 8 h. After cooling, the mixture was poured onto ice prepared with DI water and treated with 100 mL of hydrogen peroxide. The final suspension was sonicated for half an hour and centrifuged at a high speed for 1 h. The separated solid containing graphene oxide nanosheets was washed by ethanol and diluted HCl solution. Finally, the black product(GO) was hybridized with the prepared metal chalcogenide nanoparticles in order to increase the surface area and electrical conductivity.

### Modification of glassy carbon electrode with Bi_2_S_3_/GO and Bi_2_S_3_@CuS/GO

Herein, equal amounts of GO were mixed separately with Bi_2_S_3_ and *Bi*_*2*_*S*_*3*_*@CuS* nanocomposites. To each mixture, 1 mL of tetrahydrofuran (THF) and a small amount of polyvinyl chloride (PVC) were added to improve adhesion to the glassy carbon electrode surface. The resulting mixtures were sonicated separately for 20 min in a water bath. For electrode modification, 10 µL of each hybrid suspension was drop-cast onto the surface of a bare GCE and allowed to dry at room temperature for 30 min.

## Results and discussion

### Study of XRD patterns, EDX analysis and SEM images of Bi_2_S_3_ and Bi_2_S_3_@CuS nanocomposites

**Fig. 1:**
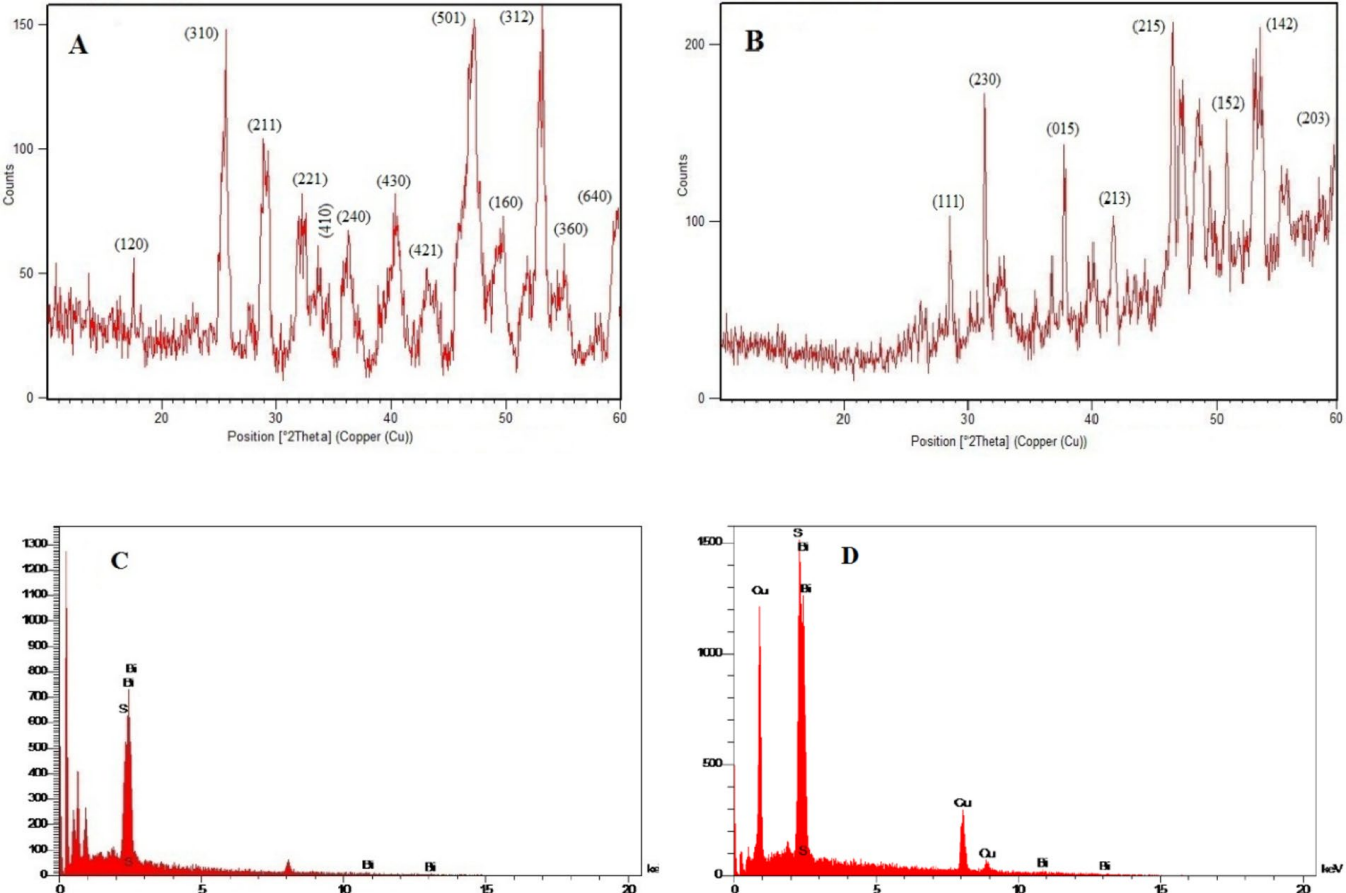
XRD pattern of Bi_2_S_3_ (**A**), Bi_2_S_3_@CuS (**B**), EDX analysis of Bi_2_S_3_ (**C**) and Bi_2_S_3_@CuS (**D**).

X-ray Diffraction pattern of the synthetic Bi_2_S_3_ has been shown in Fig. 1A. Here, the main diffraction peaks were related to orthorhombic phases. As can be seen, no impurities were detected in the structure of the synthetic compound. The sharp, narrow peaks indicate high crystallinity. The presence of the index peaks at the positions of 25º, 29 º, 32 º, 40.5 º, 47.5 º and 53.5 º confirms the structure of Bi_2_S_3_, consistent with JCPDS card number 17–0320. Figure 1B shows the X-ray diffraction spectrum of Bi_2_S_3_@CuS. In addition to the bismuthinite peaks, diffraction peaks at 28.5°, 32.6°, 48.6°, and 59.8° correspond to the CuS structure in the hodrushite phase (JCPDS card number 06–0464).

X-ray energy diffraction spectroscopy (EDX) was performed on the synthetic Bi_2_S_3_ and Bi_2_S_3_@CuS. The EDX elemental analysis diagram obtained from the synthetic metal chalcogenide, Bi_2_S_3_, is shown in Fig. 1C.Accordingly, the presence of bismuth and sulfur in the structure of the prepared material was confirmed. Figure 1D shows the results of EDX elemental analysis of Bi_2_S_3_@CuS nanocomposite. Obviously, the peaks related to bismuth, copper and sulfur elements are proof of the successful synthesis of the nanomaterial.

**Fig. 2:**
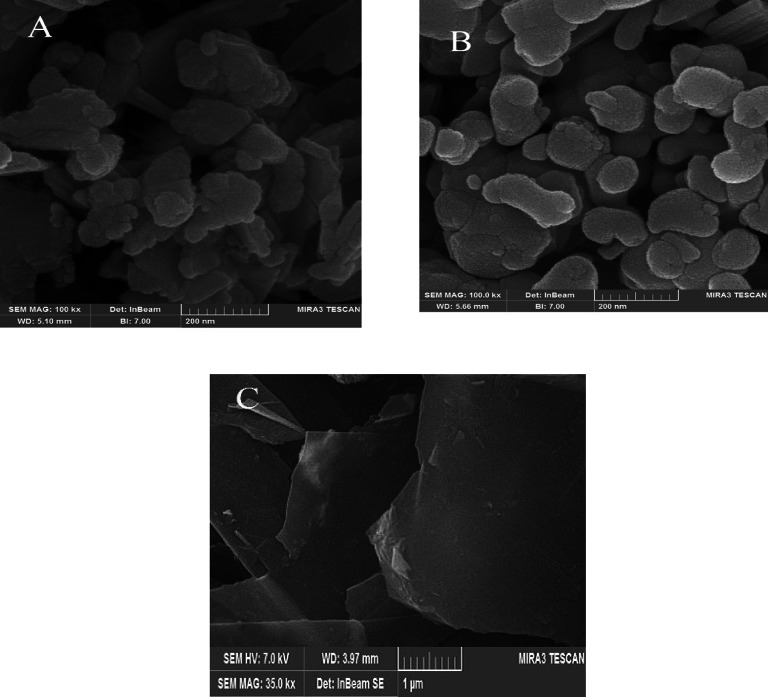
SEM images of Bi_2_S_3_ (**A**), Bi_2_S_3_@CuS (**B**) and graphene oxide nanosheets (**C**).

Figure 2A shows the SEM image of the Bi_2_S_3_ nanocomposite revealing a coral-like morphology at the nanoscale. Also, Fig. 2B presents the morphology of Bi_2_S_3_@CuS as a two-component nanocomposite, which also exhibits nanoscale features. Figure 2C displays the synthesized graphene oxide nanosheets.

### Electrochemical studies

#### Investigating the behavior of various electrodes in absence and presence of acetamiprid at different pHs

**Fig. 3:**
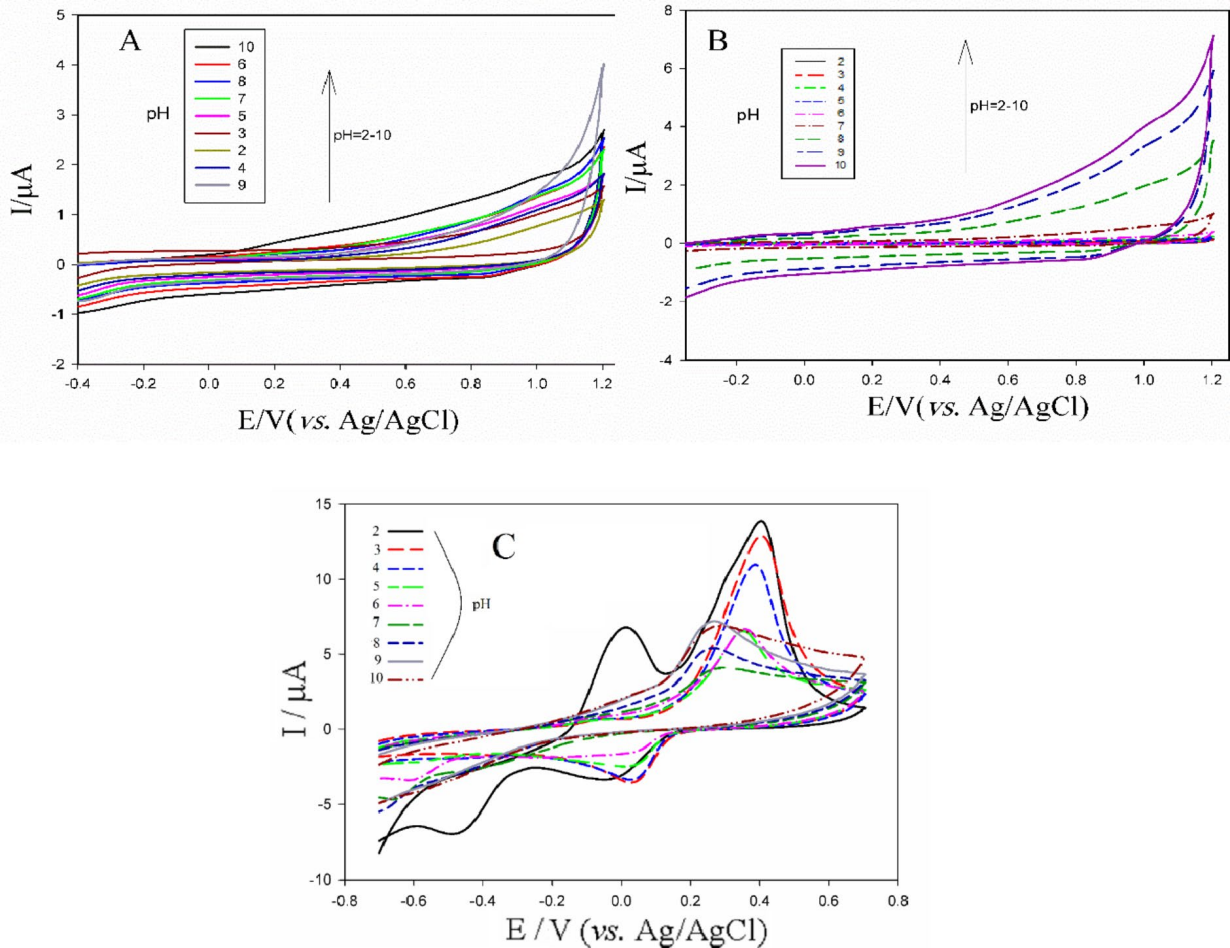
CVs illustrating electrochemical behavior of: the bare glassy carbon electrode in presence of 0.5mM of acetamiprid (**A**), Bi_2_S_3_/GO-GCE (**B**) and Bi_2_S_3_@CuS/GO(C), both in absence of acetamiprid, in various pHs (supplied by PBS). The potential scan rate was equal to 100 mV/s and the solution contained 0.1 M of KCl as supporting electrolyte.

Figure 3A shows the cyclic voltammograms(CVs) of the bare glassy carbon electrode (unmodified GCE) inserted in PBS solutions at various pH values in the presence of 0.5 mM of acetamiprid. Across a wide potential range, no electrochemical interaction between the bare GCE and acetamiprid was observed at any of the tested pH values. The only significant observation was an increase in non-faradaic current with increasing pH.

Figure 3B presents the CVs of the Bi_2_S_3_/GO modified GCE inserted in PBS solutions at various pH values without acetamiprid. Similar to the bare GCE, no oxidation or reduction processes attributable to the Bi_2_S_3_/GO –GCE were observed at any pH.

Figure 3C shows the CVs of the Bi_2_S_3_@CuS /GO modified GCE in PBS solutions at various pH values without acetamiprid. As can be seen, the copper existed in the two-component Bi_2_S_3_@CuS nanocomposite structure revealed an anodic peak near 0 V, whit the highest peak current at pH = 2. Hereon, the first oxidation peak that appeared at approximately 0V can be attributed to the oxidation of Cu^0^ to Cu^+1^ and the second oxidation peak at about 0.39 V should be related to the oxidation process of Cu^+1^ to Cu^+2^. The corresponding reduction peaks appeared at zero and − 0.45 volts, respectively. As the pH increased, the anodic peak related to the oxidation of Cu^0^ to Cu^+1^ clearly was diminished, which indicates the instability of the copper ion in this oxidation state.

**Fig. 4:**
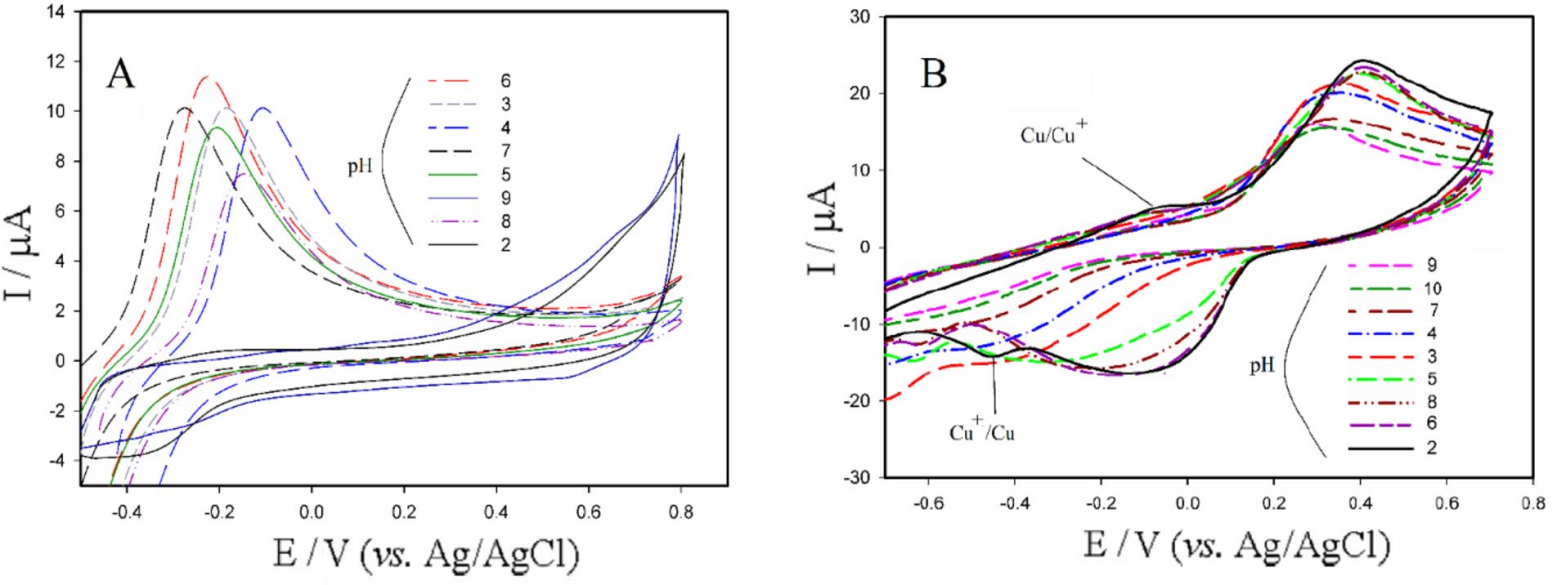
CVs related to the behavior of the electrodes modified by Bi_2_S_3_/GO (**A**) and Bi_2_S_3_@CuS/GO (**B**) in presence of 0.5 mM of acetamiprid in various pH values. The potential scan rate was equal to 100 mV/s and the solution contained 0.1 M of KCl as supporting electrolyte.

Figure 4A represents cyclic voltammograms related to Bi_2_S_3_/GO-GCE inserted in 0.5 mM of acetamiprid at various pH values. As can be seen, the highest oxidation current response corresponded to acetamiprid electrooxidation was observed at pH = 6. Here, the acetamiprid exhibited a wide pH range for the current response which is advantage for the fabricated sensor based on Bi_2_S_3_/GO. The potential values of anodic peaks across the pH range have shifted from − 0.293 to −0.108 V. In the high acidic and alkaline settings (pH 2 and 9), the acetamiprid oxidation process was hindered.

Subsequently, cyclic voltammograms, illustrating the behavior of Bi_2_S_3_@CuS/GO-GCE at various pH values _in_ the presence of 0.5 mM of acetamiprid, were recorded (Fig. 4B). At the pH = 2, weak oxidation and reduction peaks can be seen around − 0.048 and − 0.46 V respectively, likely due to the surface adsorption of acetamiprid influencing the Cu^+1^/Cu^0^ redox couple. At the other pH values, the weak peaks have been disappeared, which confirms the disturbance of the Cu^0^/Cu^+1^ redox process. However, the effect of acetamiprid on the Cu^+1^/Cu^+2^ redox process was well seen in the range of 0.4 V (oxidation) and − 0.1 V (reduction), which led to increase in the current response in the most of pHs especially acidic setting (pH = 2). Therefore, the pH 2 was selected as the optimal pH for the quantitative analysis of acetamiprid using Bi_2_S_3_@CuS/GO-GCE.

#### *Study of red/ox behavior pattern of acetamiprid on the surface of the Bi*_*2*_*S*_*3*_*/GO and Bi*_*2*_*S*_*3*_*@CuS/GO modified glassy carbon electrodes*.

**Fig. 5:**
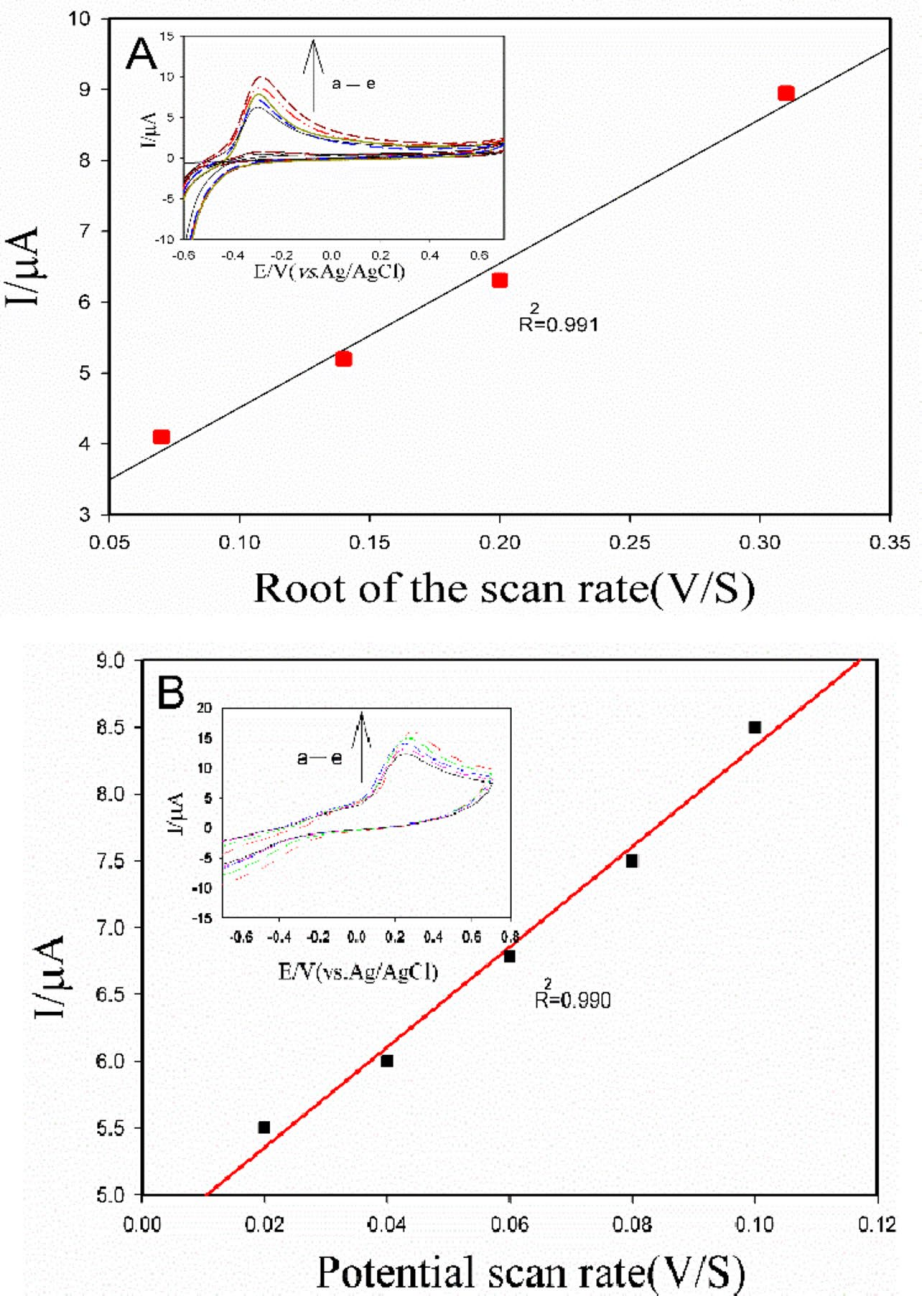
the plots extracted from CVs related to the behavior of the Bi 2 S 3 /GO-GCE (**A**) and Bi 2 S 3 @CuS/GO-GCE (**B**) inserted in PBS solution (pH = 7), in presence of 0.5 mM of acetamiprid at the scan rates of 5,10,20,40 and 100mV/s and 20,40,60,80 and 100 mV/s(a→e), respectively. (inset: the corresponded CVs). The solution was contained 0.1 M of KCl as the supporting electrolyte.

Herein, the Bi_2_S_3_/GO-GCE was inserted in a buffer solution of 0.5 mM acetamiprid (pH = 7). Cyclic voltammograms were recorded with different scan rates (ranging from 5 to 100 mV/s). Figure 5A shows the relationship between the anodic peak current and the square root of the scan rates (v/s). A linear correlation is observed between the current and the square root of scan rate within the range of 0.05 to 0.1 V/s. According to the obtained results, the redox behavior of acetamiprid on the surface of the Bi_2_S_3_/GO electrode is in agree with the diffusion pattern.

Fig 5 B. illustrates the linear correlation between the anodic peak currents and the scan rates related to recorded CVs at the same conditions using the Bi_2_S_3_@CuS/GO-GCE at the potential scan rate range of 20 to 100mV/s. This linear correlation indicates a non-diffusion-controlled, or surface-controlled, electron transfer process at the surface of the Bi_2_S_3_@CuS/GO-GCE. 

#### Investigation of redox processes mechanism of acetamiprid on the surface of the Bi_2_S_3_/GO-GCE and Bi_2_S_3_@CuS/GO-GCE

**Fig. 6:**
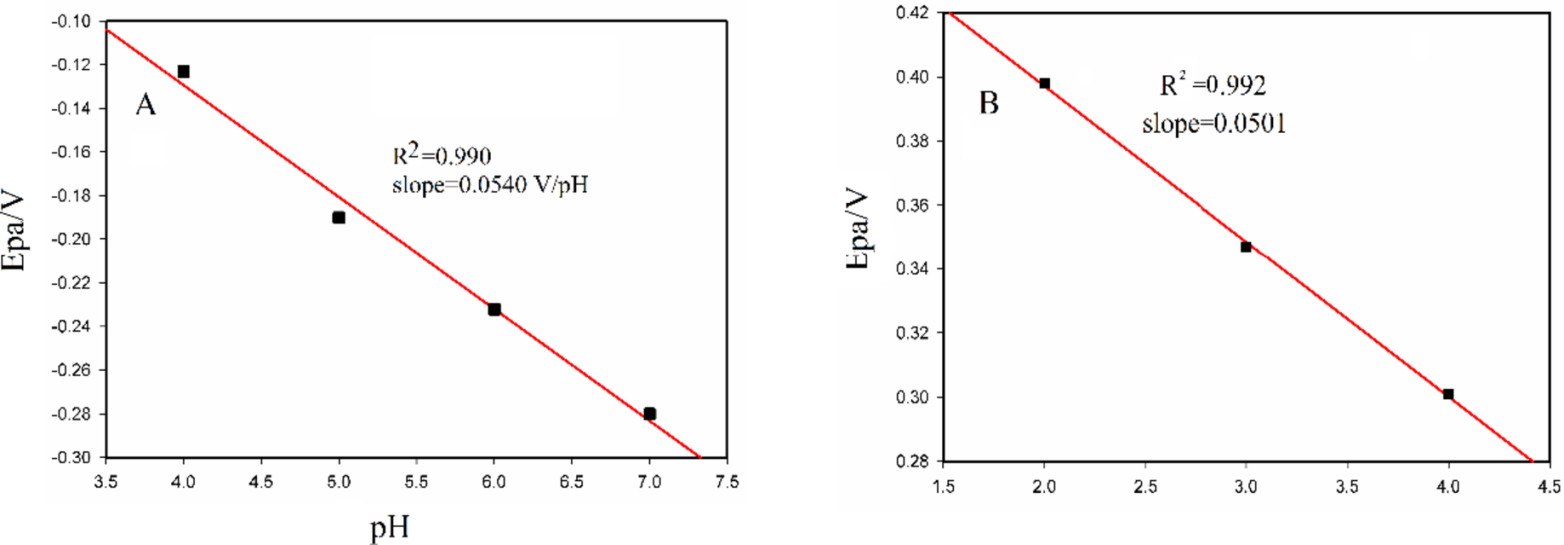
diagram of the peak potential (E_Pa_) versus pH corresponded to the modified Bi_2_S_3_/GO-GCE (**A**) and Bi_2_S_3_@CuS/GO-GCE (**B**). The electrodes were inserted in buffer solutions of 0.5 mM of acetamiprid. The potential scan rate was equal to 100 mV/s and the solution contained 0.1 M of KCl as supporting electrolyte.

To investigate the redox mechanism of the acetamiprid on the surface of the two case study electrodes, the CVs of Fig. 4A and B were analyzed. In this regard, the peak potential (E_P_) was plotted against the corresponded pH values (Fig. 6A and B). A linear correlation was found between the peak potential (E_P_) and the pHs (from 4 to 7) for the case Bi_2_S_3_/GO-GCE. The slope of the line was calculated equal to 0.0540 V/pH which is very close to the Nernstian slope (0.0591 V/pH). This proximity suggests that an equal number of electrons and protons are exchanged during the redox reaction. Similarly, a linear relationship between the anodic peak potential (E_Pa_) and pH (from 2 to 4) was found for the Bi_2_S_3_@CuS/GO-GCE. According to the calculated slope (0.0501 V/pH), the number of exchanged electrons and protons are equivalent too.

Based on the obtained results and authentic reports^65,66^, it can be concluded that acetamiprid decomposes upon oxidation on the surface of the two modified electrodes. Initially, water molecules oxidized to radical hydroxide (OH°), accompanied by the release of oxygen:

1. Nanocomposite + H2O → (nanocomposite) (OH°)ads + H+ + e
2. (Nanocomposite) (OH°)ads → nanocomposite + $\frac{1}{2}{O_2}$ + H+ + e

Concurrently, the following reaction occurs:

(nanocomposite) (OH°) + Acetamiprid →CO_2_ + nanocomposite + xH^+^ + xe^−^.

It appears that the process of oxygen release interferes with the oxidation process of acetamiprid. Therefore, modifying the electrode with Bi_2_S_3_ and Bi_2_S_3_@CuS should enhance the current response of acetamiprid electrooxidation by hindering oxygen production.

## Calculation of electrochemically active surface area (ESA) for Bi_2_S_3_/GO-GCE and Bi_2_S_3_@CuS/GO-GCE

One reliable methods for calculating the electrochemically surface area (ESA) is using the Randles-Sevcik equation:

$$I_{p}=2.69\times10^{5} n^{3/2}AC*D^{1/2} \upsilon^{1/2}$$

Where, *n* is the number of transferred electrons corresponded to redox process of the K_3_Fe(CN)_6_ solution as the probe on the surface of the electrodes (*n* = 1). *A* is the active surface area(cm^2^), C* represents the concentration of the used probe (mM) and *D* is the diffusion coefficient of K_3_Fe(CN)_6_, which is equal to 6.7 × 10^−6^ cm^2^/s^67^. Finally, *ʋ* stands for the potential scan rate (v/s).

To determine the ESA, the Bi_2_S_3_/GO-GCE and Bi_2_S_3_@CuS/GO-GCE were individually immersed in 1 mM of K_3_Fe(CN)_6_ PBS solution (pH = 7). Cyclic voltammograms were recorded at the scan rates of 0.2, 0.4, 0.6, 0.8 and 1 v/s. The anodic peak currents were extracted and plotted against the square root of the scan rate.

By calculation the slope of the resulting lines, the electrochemical active surface areas were determined to be 0.278 and 0.355cm^2^ for Bi_2_S_3_/GO-GCE and Bi_2_S_3_@CuS/GO-GCE, respectively. Compared to the geometric surface area of the bare glassy carbon electrode (*r* = 2 mm, area = 0.0314 cm^2^) the modification significantly increased the ESA. Also, the increase in the surface area of the case Bi_2_S_3_@CuS/GO-GCE was noticeably greater than Bi_2_S_3_/GO-GCE, which is expected due to the presence of copper in the nanocomposite structure.

According to the obtained results, incorporating copper atoms into the structure of bismuth chalcogenide, increases the level of electrical conductivity and improves the current response and sensitivity of the acetamiprid measurement. Over here, the inclusion of a transition metal chalcogenide to the bismuthinite structure creates a synergistic effect for electrocatalysis of the acetamiprid electrooxidation (as discussed previously). Consequently, the Bi_2_S_3_@CuS/GO-GCE was selected for subsequent quantitative analysis of acetamiprid using differential pulse voltammetry (DPV).

### Quantitative analysis of acetamiprid by Bi_2_S_3_@CuS/GO-GCE using differential pulse voltammetry(DPV)

**Fig. 7:**
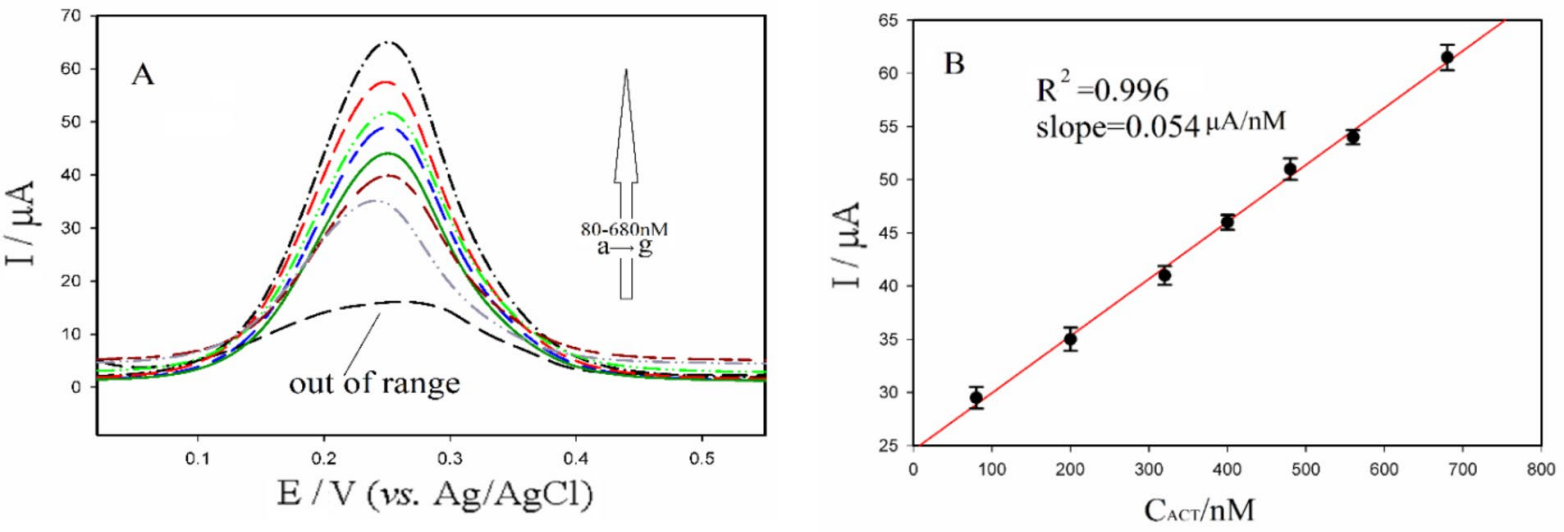
DPVs corresponding to various concentrations of acetamiprid (80,200, 320, 400, 480, 560 and 680nM (a→g)) under optimal condition (**A**) and the extracted calibration curve (**B**).

Subsequently, varying concentrations of acetamiprid in the nanomolar range were prepared and analyzed using differential pulse voltammetry (DPV). The Bi₂S₃@CuS/GO-GCE was placed in a pH 2 phosphate buffered saline (PBS) solution containing different acetamiprid concentrations under the previously established conditions. The resulting voltammograms are displayed in Fig. 7A. Figure 7B shows the linear relationship between the current response and the applied acetamiprid concentrations. The electrode demonstrated a linear response across a broad concentration range from 80 to 680 nM (R² = 0.996). The slope of the calibration curve, representing the sensitivity of the method, was calculated to be 0.054 µA/nM. These findings highlight the potential of the Bi₂S₃@CuS/GO-GCE for trace-level acetamiprid analysis.

The detection limit of the method was determined by performing seven replicate measurements of a specific acetamiprid solution buffered in PBS using the Bi₂S₃@CuS/GO-GCE. The limit of detection (LOD) was calculated using the Eq. 3Sb/b, where Sb represents the standard deviation of the replicate blank measurements, and b represents the slope of the calibration curve. Using this equation, the LOD was determined to be 4.1 nM, confirming the very low detection limit of the proposed sensor.

### Reproducibility and stability of the Bi_2_S_3_@CuS/GO-GCE

To assess the reproducibility of the fabricated sensor, five separately modified electrodes were used to analyze a buffer solution containing 200 nM acetamiprid via differential pulse voltammetry (DPV). The relative standard deviation (RSD%) of the obtained results was calculated to be 2.37%, indicating acceptable sensor reproducibility. Two aspects of stability were also studied by Bi_2_S_3_@CuS/GO-GCE. First, a typical modified electrode was kept at room temperature for two weeks and a known concentration of acetamiprid was measured by differential pulse voltammetry. The decrease in the current response (peak current) after two weeks was determined to be less than 5%:

$$\frac{{{I_P}({{14}^{th}}day)}}{{{I_P}({1^{th}}day)}} \times 100=97.3$$

Furthermore, 30 voltammetric measurements of a certain buffered solution of acetamiprid (200 nM) were performed by a typical modified electrode. Here, the peak current drop was calculated to be less than 5% too.

The performance of the fabricated Bi_2_S_3_@CuS/GO-GCE was compared with some reported strategies used for acetamiprid detection in recent years and presented (Table 1). While some aptamer-based methods offer a wider linear range, this simple electrochemical strategy provides a lower detection limit, greater simplicity, and easier handling compared to other methods.

**Fig. 8:**
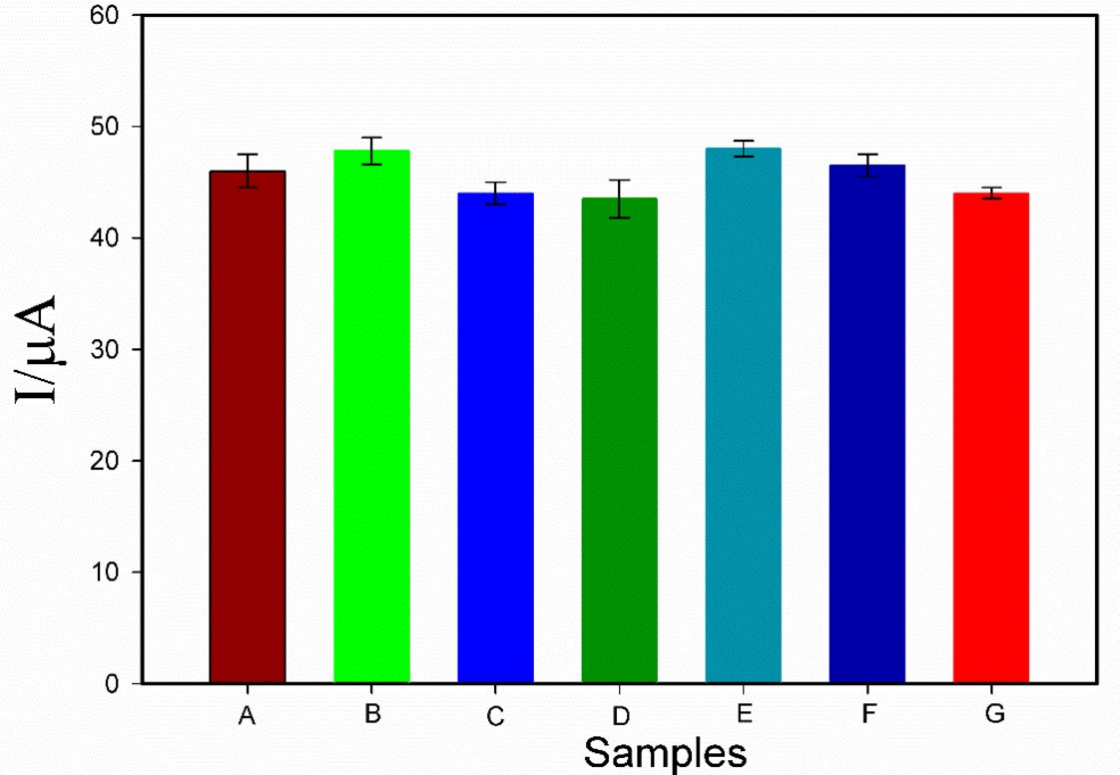
DPV current responses created by Bi_2_S_3_@CuS/GO-GCE related to the buffered solution of 400nM of acetamiprid(**A**) and the same solution with 50nM of: carbaryl(**B**), chlorpyrifos(**C**), imidacloprid(**D**), phoxim(**E**), dichlorvos(**F**) and thiacloprid(**G**).

For evaluating the selectivity of the Bi_2_S_3_@CuS/GO-GCE for detection of acetamiprid, various interfering pesticides to a buffered solution containing 400 nM acetamiprid. The results (Fig. 8) demonstrate minimal interference from these coexisting substances relative to the acetamiprid signal.

**Table 1:** Comparison of the present work with recent methods for acetamiprid detection.

method	Linear range	LOD	Ref.
Colorimetric detection based on the enhanced peroxidase-like activity of gold nanoparticles	10–50 µg/l	1.02 µg/l	^68^
Aptamer-Controlled Reversible Inhibition of Gold Nanozyme Activity	100–10,000 µg/l	100 µg/l	^69^
Colorimetric detection method by fine-tuning aptamer length	--------	400nM	^70^
Aptamer-based fluorescent screening assay via inner filter effect of gold nanoparticles on the fluorescence of CdTe quantum dots	11–223 µg/l	1.6 µg/l	^71^
QD-aptamer as a donor for a FRET-based chemosensor	10-10000nM	20	^72^
Impedance spectroscopy-based aptasensor	5-600nM	1nM	^73^
A disposable electrochemical DNA aptasensor	56–446 µg/l	19 µg/l	^74^
A sensor based on TiO_2_ photocatalysis for degradation of acetamiprid	10-2000nM	200nM	^75^
High-Performance Liquid Chromatography with Diode-Array Detection	----	45nM	^76^
A colorimetric method based on the aggregation of gold nanoparticles	6.6 × 10^−7^ − 6.6 × 10^−5^M	44nM	^77^
Solid-phase extraction and liquid chromatography–mass spectrometry	20-2200nM	90nM	^78^
Current work	80-680nM 17.8–151 µg/l	4.1nM 0.91 µg/l	-----

### Real sample analysis

An electrochemical sensor demonstrates its value when it operates effectively without chemical or electrochemical interference in real samples, even within complex matrices. Here, a typical Bi_2_S_3_@CuS/GO-GCE was employed to determine acetamiprid in tomato samples and well water. First, the tomato samples were grated and filtered, and the resulting filtrate was collected and transferred to a 100 ml volumetric flask, and the pH was adjusted by phosphate buffer (pH = 2). Similarly, three samples of a local well water were buffered to a pH of 2. Specific amounts of acetamiprid were then added to these prepared samples, and the analyte concentration was determined using differential pulse voltammetry. The recovery percentages of acetamiprid obtained with this electrochemical sensor are presented in Table 2.

**Fig. 9:**
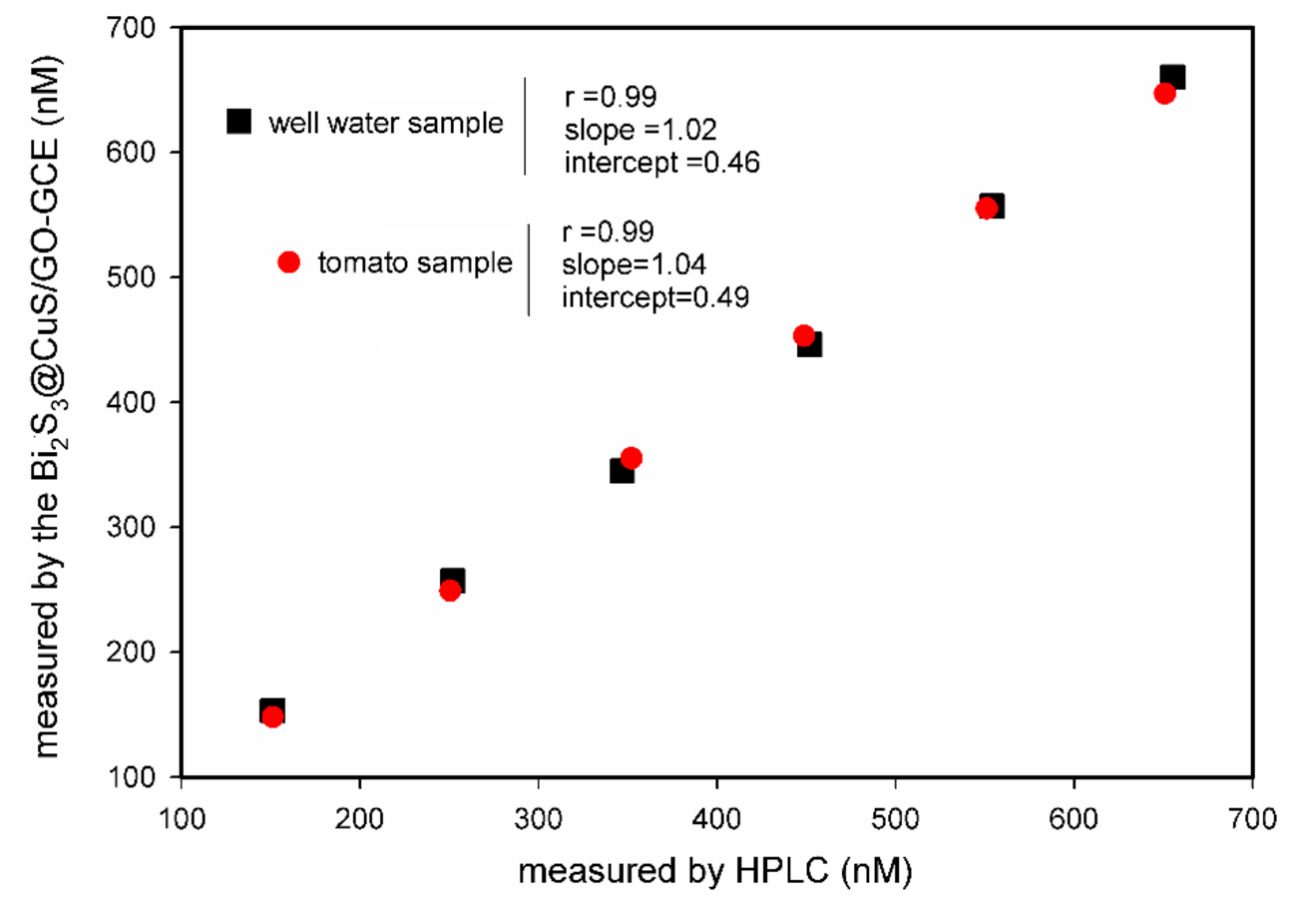
Correlation between the results obtained from the current method (y-axis) and the reference method(X-axis) for detection six different concentrations of acetamiprid (150,250, 350,450,550 and 650nM), HPLC conditions: Column: C18 reverse-phase, detection: UV at 245 nm, mobile phase: acetonitrile: water (65:35 V/V).

High-performance liquid chromatography (HPLC) is a well-established reference technique and one of the most common and reliable methods for measuring various physiological compounds. Herein, the same concentrations of the acetamiprid were measured using the Bi_2_S_3_@CuS/GO-GCE. Figure 9 presents the correlation analysis between the current method and the standard HPLC method for the two studied samples. The correlation coefficient (r) demonstrates strong linearity in both cases. Moreover, the slope and intercept values are very close to one and zero, respectively. As well, the calculated bias of the data is very close to zero for both sample analyses. Overall, the method was validated as an unbiased and accurate approach alternative to HPLC.

**Table 2:** The results related to real sample analysis of acetamiprid by Bi_2_S_3_@CuS/GO-GCE in the optimized conditions.

Sample	No.	Spiked acetamiprid (nM)	Detected acetamiprid(nM)	Recovery (%)	RSD(%)
tomato	1	100	105.0	105.0	2.65
	2	300	288.0	96.0	3.07
	3	600	572.0	95.0	2.44
well water	1	100	102.2	102.2	1.92
	2	300	304.5	101.5	1.80
	3	600	587.0	97.8	2.43

## Conclusion

Bismuthinite and bismuthinite @copper sulphide (Bi_2_S_3_ and Bi_2_S_3_@CuS) were hydrothermally synthesized and developed for electrochemical sensing of acetamiprid (Mospilan) pesticide in ultra-trace levels in real samples. The suggested Bi_2_S_3_@CuS/GO-GCE sensor was applied as an alternative strategy for complicated enzymatic and aptamer based methods. While the Bi_2_S_3_/GO-GCE demonstrated a current response to acetamiprid across a wide pH range, the results indicate that Bi_2_S_3_@CuS nanoelectrocatalyst is more resistant to the oxygen evolution process during acetamiprid electrooxidation. This resistance leads to a significantly enhanced current response for acetamiprid measurement. The detection limit for acetamiprid using Bi_2_S_3_@CuS/GO-GCE was estimated about 4.1nM. The fabricated sensor also exhibits high sensitivity and repeatability. Furthermore, the Bi_2_S_3_@CuS/GO-GCE demonstrated good recovery percentages for acetamiprid detection in real samples.

## Data Availability

The data generated or analyzed during the current study is available upon request from the corresponding author.
